# Supplementary material for: Health status of free-ranging ring-necked pheasant chicks (Phasianus colchicus) in North-Western Germany
Source: PLoS One. 2020 Jun 16;15(6):e0234044. doi: 10.1371/journal.pone.0234044 (PMC7297342; doi:10.1371/journal.pone.0234044)
Supplement: S1 Table — Highlighted in bold are the substances found in the pheasant samples. (DOCX) [file pone.0234044.s001.docx]

**S1 Table: Toxicological investigation of substances and limits of detection. Highlighted in bold are the substances found in the pheasant samples.**

| **Pesticide screening using GC / MS for complex foods** | | **Pesticide screening using LC / MS / MS in high-fat foods** | | | **Glyphosate, AMPA in food** | | |
| --- | --- | --- | --- | --- | --- | --- | --- |
| **Parameter** | Limit of quantification | Parameter | Limit of quantification | Parameter | | Limit of quantification |  |
| 2,4,5-T-methylester | 0.02 mg/kg | 2,4'-Formoxylidid | 0.01 mg/kg | Aminomethylphosphonic acid (AMPA) | | 0.01 mg/kg |  |
| 2,4-D-methylester | 0.1 mg/kg | 3-Hydroxycarbofuran | 0.01 mg/kg | Glyphosate | | 0.01 mg/kg |  |
| Acetochlor | 0.02 mg/kg | 6-Chlor-3-Phenylpyridazin-4-ol (Pyridatmetabolit) | 0.01 mg/kg |  | |  |  |
| Aclonifen | 0.02 mg/kg | Abamectin | 0.01 mg/kg |  | |  |  |
| Acrinathrin | 0.01 mg/kg | Acephat | 0.01 mg/kg |  | |  |  |
| Alachlor | 0.1 mg/kg | Acetamiprid | 0.01 mg/kg |  | |  |  |
| Aldrin | 0.005 mg/kg | Acetochlor | 0.02 mg/kg |  | |  |  |
| Allethrin | 0.03 mg/kg | Alachlor | 0.01 mg/kg |  | |  |  |
| Amidithion | 0.03 mg/kg | Aldicarb | 0.01 mg/kg |  | |  |  |
| Azaconazol | 0.02 mg/kg | Aldicarb-sulfon | 0.01 mg/kg |  | |  |  |
| Azinphos-ethyl | 0.01 mg/kg | Aldicarb-sulfoxid | 0.01 mg/kg |  | |  |  |
| Azinphos-methyl | 0.01 mg/kg | Ametoctradin | 0.01 mg/kg |  | |  |  |
| Azoxystrobin | 0.01 mg/kg | Ametryn | 0.01 mg/kg |  | |  |  |
| Benfluralin | 0.005 mg/kg | Amidosulfuron | 0.01 mg/kg |  | |  |  |
| Benoxacor | 0.01 mg/kg | Aminocarb | 0.01 mg/kg |  | |  |  |
| Benzoylprop-ethyl | 0.01 mg/kg | Amitraz | 0.01 mg/kg |  | |  |  |
| Bifenthrin | 0.005 mg/kg | Ancymidol | 0.01 mg/kg |  | |  |  |
| Binapacryl | 0.02 mg/kg | Atrazin | 0.01 mg/kg |  | |  |  |
| Bitertanol | 0.1 mg/kg | Atrazin, desethyl- | 0.01 mg/kg |  | |  |  |
| Boscalid | 0.02 mg/kg | Azaconazol | 0.01 mg/kg |  | |  |  |
| Bromcyclen | 0.01 mg/kg | Azamethiphos | 0.01 mg/kg |  | |  |  |
| Bromfenvinphos | 0.01 mg/kg | Aziprotryn | 0.01 mg/kg |  | |  |  |
| Bromophos-ethyl | 0.005 mg/kg | Azoxystrobin | 0.01 mg/kg |  | |  |  |
| Bromophos-methyl | 0.005 mg/kg | Benalaxyl | 0.01 mg/kg |  | |  |  |
| Brompropylat | 0.01 mg/kg | Bendiocarb | 0.01 mg/kg |  | |  |  |
| Buprofezin | 0.05 mg/kg | Benfuracarb | 0.01 mg/kg |  | |  |  |
| Butachlor | 0.1 mg/kg | Benodanil | 0.01 mg/kg |  | |  |  |
| Butralin | 0.01 mg/kg | Bensulfuron methyl | 0.01 mg/kg |  | |  |  |
| Cadusafos | 0.01 mg/kg | Benthiavalicarb, isopropyl- | 0.01 mg/kg |  | |  |  |
| Captafol | 0.02 mg/kg | Bitertanol | 0.01 mg/kg |  | |  |  |
| Captan | 0.02 mg/kg | Boscalid | 0.01 mg/kg |  | |  |  |
| Carbophenothion | 0.005 mg/kg | Bromacil | 0.01 mg/kg |  | |  |  |
| Carbophenothion-methyl | 0.01 mg/kg | Bromuconazol (Summe) | 0.01 mg/kg |  | |  |  |
| Carfentrazon-ethyl | 0.01 mg/kg | Bupirimat | 0.01 mg/kg |  | |  |  |
| Chinomethionat | 0.01 mg/kg | Buprofezin | 0.01 mg/kg |  | |  |  |
| Chlorbensid | 0.005 mg/kg | Butachlor | 0.02 mg/kg |  | |  |  |
| Chlordan, cis- | 0.01 mg/kg | Butocarboxim-sulfoxid | 0.01 mg/kg |  | |  |  |
| Chlordan, oxy- | 0.01 mg/kg | Butoxycarboxim | 0.01 mg/kg |  | |  |  |
| Chlordan, trans- | 0.01 mg/kg | Buturon | 0.01 mg/kg |  | |  |  |
| Chlorethoxyfos | 0.01 mg/kg | Carbaryl | 0.01 mg/kg |  | |  |  |
| Chlorfenapyr | 0.005 mg/kg | Carbendazim | 0.01 mg/kg |  | |  |  |
| Chlorfenson | 0.005 mg/kg | Carbofuran | 0.01 mg/kg |  | |  |  |
| Chloridazon | 0.1 mg/kg | Carbosulfan | 0.01 mg/kg |  | |  |  |
| Chlormephos | 0.005 mg/kg | Carboxin | 0.01 mg/kg |  | |  |  |
| Chloroneb | 0.1 mg/kg | Chlorantraniliprol | 0.01 mg/kg |  | |  |  |
| Chlorpropylat | 0.005 mg/kg | Chlorbromuron | 0.01 mg/kg |  | |  |  |
| Chlorpyrifos (-ethyl) | 0.005 mg/kg | Chlorfluazuron | 0.01 mg/kg |  | |  |  |
| Chlorpyriphos-methyl | 0.005 mg/kg | Chloridazon | 0.01 mg/kg |  | |  |  |
| Chlorthal-dimethyl | 0.005 mg/kg | Chloroxuron | 0.01 mg/kg |  | |  |  |
| Chlorthalonil | 0.005 mg/kg | Chlorpropham | 0.05 mg/kg |  | |  |  |
| Chlorthion | 0.01 mg/kg | Chlorsulfuron | 0.01 mg/kg |  | |  |  |
| Chlorthiophos | 0.005 mg/kg | Chlortoluron | 0.01 mg/kg |  | |  |  |
| Chlozolinate | 0.005 mg/kg | Cinidon-ethyl | 0.05 mg/kg |  | |  |  |
| Cinidon-ethyl | 0.05 mg/kg | Cinosulfuron | 0.01 mg/kg |  | |  |  |
| Clodinafop-propargyl | 0.05 mg/kg | Clethodim | 0.01 mg/kg |  | |  |  |
| Coumaphos | 0.005 mg/kg | Clodinafop-propargyl | 0.01 mg/kg |  | |  |  |
| Crotoxyphos | 0.01 mg/kg | Clofentezin | 0.01 mg/kg |  | |  |  |
| Cyanofenphos | 0.005 mg/kg | Clomazon | 0.01 mg/kg |  | |  |  |
| Cyanophos | 0.01 mg/kg | Clothianidin | 0.01 mg/kg |  | |  |  |
| Cyfluthrin | 0.005 mg/kg | Crufomat | 0.01 mg/kg |  | |  |  |
| Cyhalothrin, lambda- | 0.005 mg/kg | Cyanazin | 0.01 mg/kg |  | |  |  |
| Cypermethrin | 0.005 mg/kg | Cyazofamid | 0.01 mg/kg |  | |  |  |
| Cyphenothrin | 0.01 mg/kg | Cymoxanil | 0.02 mg/kg |  | |  |  |
| Cyproconazol | 0.05 mg/kg | Cyproconazol | 0.01 mg/kg |  | |  |  |
| DDD, o,p- | 0.005 mg/kg | Cyprodinil | 0.01 mg/kg |  | |  |  |
| DDD, p,p- | 0.005 mg/kg | Cyprofuram | 0.01 mg/kg |  | |  |  |
| DDE, o,p- | 0.005 mg/kg | Cyromazin | 0.01 mg/kg |  | |  |  |
| DDE, p,p'- | 0.005 mg/kg | Demeton | 0.01 mg/kg |  | |  |  |
| DDT, o,p'- | 0.005 mg/kg | Demeton-S-methyl-sulfon | 0.01 mg/kg |  | |  |  |
| DDT, p,p- | 0.005 mg/kg | Desmedipham | 0.01 mg/kg |  | |  |  |
| Deltamethrin | 0.01 mg/kg | Desmetryn | 0.01 mg/kg |  | |  |  |
| Diallat | 0.01 mg/kg | Diazinon | 0.01 mg/kg |  | |  |  |
| Diazinon | 0.01 mg/kg | Dichlorvos | 0.01 mg/kg |  | |  |  |
| Dibrombenzophenon, p,p- | 0.01 mg/kg | Diclobutrazol | 0.01 mg/kg |  | |  |  |
| Dicapthon | 0.01 mg/kg | Diethofencarb | 0.01 mg/kg |  | |  |  |
| Dichlobenil | 0.02 mg/kg | Diethyltoluamid | 0.01 mg/kg |  | |  |  |
| Dichlofenthion | 0.005 mg/kg | Difenoconazol | 0.01 mg/kg |  | |  |  |
| Dichlofluanid | 0.01 mg/kg | Difenoxuron | 0.01 mg/kg |  | |  |  |
| Dichlorbenzophenon, o,p- | 0.01 mg/kg | Diflubenzuron | 0.01 mg/kg |  | |  |  |
| Dichlorbenzophenon, p,p- | 0.01 mg/kg | ***Diflufenican*** | 0.01 mg/kg |  | |  |  |
| Diclofop-methyl | 0.01 mg/kg | Dimefuron | 0.01 mg/kg |  | |  |  |
| Dicloran | 0.005 mg/kg | Dimethenamid | 0.01 mg/kg |  | |  |  |
| Dicofol, p,p- | 0.01 mg/kg | Dimethomorph | 0.01 mg/kg |  | |  |  |
| Dicrotophos | 0.01 mg/kg | Dimetilan | 0.01 mg/kg |  | |  |  |
| Dieldrin | 0.01 mg/kg | Dimoxystrobin | 0.01 mg/kg |  | |  |  |
| Difenoconazol | 0.01 mg/kg | Dinotefuran | 0.02 mg/kg |  | |  |  |
| Dimethachlor | 0.1 mg/kg | Disulfoton | 0.01 mg/kg |  | |  |  |
| Dimethipin | 0.005 mg/kg | Disulfoton-sulfon | 0.01 mg/kg |  | |  |  |
| Dimethoat | 0.02 mg/kg | Disulfoton-sulfoxid | 0.01 mg/kg |  | |  |  |
| Dimethomorph | 0.05 mg/kg | Diuron | 0.01 mg/kg |  | |  |  |
| Diniconazol | 0.01 mg/kg | Emamectin (Summe) | 0.01 mg/kg |  | |  |  |
| Dinitramin | 0.01 mg/kg | ***Epoxiconazol*** | 0.01 mg/kg |  | |  |  |
| Disulfoton | 0.01 mg/kg | Ethiofencarb | 0.01 mg/kg |  | |  |  |
| Disulfoton-sulfoxid | 0.05 mg/kg | Ethiofencarb-sulfon | 0.01 mg/kg |  | |  |  |
| Ditalimphos | 0.005 mg/kg | Ethiofencarb-sulfoxid | 0.01 mg/kg |  | |  |  |
| Edifenphos | 0.02 mg/kg | Ethiprol | 0.01 mg/kg |  | |  |  |
| Endosulfan, beta- | 0.005 mg/kg | Ethofumesat-2-keto | 0.05 mg/kg |  | |  |  |
| Endosulfan-sulfate | 0.005 mg/kg | Ethoprophos | 0.01 mg/kg |  | |  |  |
| Endrin | 0.01 mg/kg | Etofenprox | 0.01 mg/kg |  | |  |  |
| EPN | 0.01 mg/kg | Famoxadon | 0.01 mg/kg |  | |  |  |
| Epoxiconazol | 0.005 mg/kg | Fenamidon | 0.01 mg/kg |  | |  |  |
| Etaconazol | 0.01 mg/kg | Fenamiphos | 0.01 mg/kg |  | |  |  |
| Ethalfluralin | 0.005 mg/kg | Fenamiphos-sulfon | 0.01 mg/kg |  | |  |  |
| Ethion | 0.005 mg/kg | Fenamiphos-sulfoxid | 0.01 mg/kg |  | |  |  |
| Ethiprol | 0.01 mg/kg | Fenarimol | 0.01 mg/kg |  | |  |  |
| Ethofumesat | 0.2 mg/kg | Fenazaquin | 0.01 mg/kg |  | |  |  |
| Ethoprophos | 0.005 mg/kg | Fenbuconazol | 0.01 mg/kg |  | |  |  |
| Etridiazol | 0.005 mg/kg | Fenhexamid | 0.01 mg/kg |  | |  |  |
| Etrimfos | 0.005 mg/kg | Fenobucarb | 0.01 mg/kg |  | |  |  |
| Famophos | 0.02 mg/kg | Fenoxaprop-ethyl | 0.01 mg/kg |  | |  |  |
| Famoxadon | 0.01 mg/kg | Fenoxycarb | 0.01 mg/kg |  | |  |  |
| Fenamidon | 0.01 mg/kg | Fenpiclonil | 0.01 mg/kg |  | |  |  |
| Fenamiphos | 0.02 mg/kg | Fenpropidin | 0.01 mg/kg |  | |  |  |
| Fenarimol | 0.02 mg/kg | Fenpropimorph | 0.01 mg/kg |  | |  |  |
| Fenbuconazol | 0.02 mg/kg | Fenpyroximat | 0.01 mg/kg |  | |  |  |
| Fenchlorazol-ethyl | 0.02 mg/kg | Fensulfothion | 0.01 mg/kg |  | |  |  |
| Fenchlorphos | 0.005 mg/kg | Fensulfothion-oxon-sulfon | 0.01 mg/kg |  | |  |  |
| Fenfluthrin | 0.01 mg/kg | Fensulfothion-oxon-sulfoxid | 0.01 mg/kg |  | |  |  |
| Fenhexamid | 0.01 mg/kg | Fensulfothion-sulfon | 0.01 mg/kg |  | |  |  |
| Fenitrothion | 0.005 mg/kg | Fenthion | 0.01 mg/kg |  | |  |  |
| Fenpiclonil | 0.05 mg/kg | Fenthion-oxon | 0.01 mg/kg |  | |  |  |
| Fenpropathrin | 0.005 mg/kg | Fenthion-oxon-sulfon | 0.01 mg/kg |  | |  |  |
| Fenpropimorph | 0.1 mg/kg | Fenthion-oxon-sulfoxid | 0.01 mg/kg |  | |  |  |
| Fenson | 0.005 mg/kg | Fenthion-sulfon | 0.01 mg/kg |  | |  |  |
| Fensulfothion | 0.01 mg/kg | Fenthion-sulfoxid | 0.01 mg/kg |  | |  |  |
| Fenvalerate (RR-/SS-Isomere) | 0.005 mg/kg | Flazasulfuron | 0.01 mg/kg |  | |  |  |
| Fenvalerate (RS-/SR-Isomere) | 0.005 mg/kg | Florasulam | 0.01 mg/kg |  | |  |  |
| Fipronil | 0.01 mg/kg | Fluazifop-P-butyl | 0.01 mg/kg |  | |  |  |
| Fipronil, desulfinyl- | 0.01 mg/kg | Fluazuron | 0.02 mg/kg |  | |  |  |
| Fipronil-sulfid | 0.01 mg/kg | Flucycloxuron | 0.01 mg/kg |  | |  |  |
| Fipronil-sulfon | 0.01 mg/kg | Fludioxonil | 0.01 mg/kg |  | |  |  |
| Flamprop-isopropyl | 0.02 mg/kg | Flufenacet | 0.01 mg/kg |  | |  |  |
| Flamprop-methyl | 0.02 mg/kg | Flufenoxuron | 0.01 mg/kg |  | |  |  |
| Flonicamid | 0.05 mg/kg | Fluometuron | 0.01 mg/kg |  | |  |  |
| Fluazifop-butyl | 0.1 mg/kg | Fluopicolid | 0.01 mg/kg |  | |  |  |
| Fluazinam | 0.02 mg/kg | Flurochloridon | 0.01 mg/kg |  | |  |  |
| Fluchloralin | 0.025 mg/kg | Flurprimidol | 0.01 mg/kg |  | |  |  |
| Flucythrinat | 0.01 mg/kg | Flusilazol | 0.01 mg/kg |  | |  |  |
| Flufenoxuron | 0.01 mg/kg | Flutriafol | 0.01 mg/kg |  | |  |  |
| Flumethrin | 0.1 mg/kg | FM-6-1 | 0.05 mg/kg |  | |  |  |
| Flumetralin | 0.005 mg/kg | Formetanat | 0.01 mg/kg |  | |  |  |
| Fluopicolid | 0.01 mg/kg | Fosthiazat | 0.01 mg/kg |  | |  |  |
| Fluorodifen | 0.005 mg/kg | Fuberidazol | 0.01 mg/kg |  | |  |  |
| Fluotrimazol | 0.01 mg/kg | Furathiocarb | 0.01 mg/kg |  | |  |  |
| Fluquinconazol | 0.01 mg/kg | Halofenozid | 0.01 mg/kg |  | |  |  |
| Flurenol-butyl | 0.01 mg/kg | Haloxyfop-ethoxyethyl | 0.01 mg/kg |  | |  |  |
| Flurochloridon | 0.02 mg/kg | Haloxyfop-methyl | 0.01 mg/kg |  | |  |  |
| Flurtamon | 0.01 mg/kg | Hexaconazol | 0.01 mg/kg |  | |  |  |
| Flusilazol | 0.1 mg/kg | Hexaflumuron | 0.05 mg/kg |  | |  |  |
| Folpet | 0.025 mg/kg | Hexazinon | 0.01 mg/kg |  | |  |  |
| Fonofos | 0.005 mg/kg | Hexythiazox | 0.01 mg/kg |  | |  |  |
| Formothion | 0.005 mg/kg | Imazalil | 0.01 mg/kg |  | |  |  |
| Genite | 0.02 mg/kg | Imibenconazol | 0.01 mg/kg |  | |  |  |
| Halfenprox | 0.005 mg/kg | Imidacloprid | 0.01 mg/kg |  | |  |  |
| Haloxyfop-ethoxyethyl | 0.01 mg/kg | Indoxacarb | 0.01 mg/kg |  | |  |  |
| Haloxyfop-methyl | 0.02 mg/kg | Iodosulfuron-methyl | 0.01 mg/kg |  | |  |  |
| HCH, alpha- | 0.005 mg/kg | Isoprocarb | 0.01 mg/kg |  | |  |  |
| HCH, beta- | 0.005 mg/kg | Isoprothiolan | 0.01 mg/kg |  | |  |  |
| HCH, delta- | 0.005 mg/kg | Isoproturon | 0.01 mg/kg |  | |  |  |
| HCH, epsilon- | 0.005 mg/kg | Isoxaben | 0.01 mg/kg |  | |  |  |
| Heptachlor | 0.005 mg/kg | Isoxaflutol | 0.01 mg/kg |  | |  |  |
| Heptachlorepoxid, cis- | 0.01 mg/kg | Lenacil | 0.01 mg/kg |  | |  |  |
| Heptachlorepoxid, trans- | 0.01 mg/kg | Linuron | 0.01 mg/kg |  | |  |  |
| Heptenophos | 0.005 mg/kg | Lufenuron | 0.05 mg/kg |  | |  |  |
| Hexachlorbenzol (HCB) | 0.005 mg/kg | Malaoxon | 0.01 mg/kg |  | |  |  |
| Hexaconazol | 0.005 mg/kg | Malathion | 0.01 mg/kg |  | |  |  |
| Indoxacarb | 0.01 mg/kg | Mandipropamid | 0.01 mg/kg |  | |  |  |
| Iodofenphos | 0.005 mg/kg | Mepanipyrim | 0.01 mg/kg |  | |  |  |
| Ioxynil-octanoat | 0.02 mg/kg | Metalaxyl | 0.01 mg/kg |  | |  |  |
| Iprobenfos | 0.01 mg/kg | Metamitron | 0.01 mg/kg |  | |  |  |
| Iprodion | 0.02 mg/kg | Metazachlor | 0.01 mg/kg |  | |  |  |
| Isazofos | 0.02 mg/kg | Metconazol | 0.01 mg/kg |  | |  |  |
| Isobenzan | 0.005 mg/kg | Methabenzthiazuron | 0.01 mg/kg |  | |  |  |
| Isocarbofos | 0.005 mg/kg | Methacrifos | 0.01 mg/kg |  | |  |  |
| Isodrin | 0.005 mg/kg | Methamidophos | 0.01 mg/kg |  | |  |  |
| Isofenphos | 0.005 mg/kg | Methidathion | 0.01 mg/kg |  | |  |  |
| Isofenphos-methyl | 0.005 mg/kg | Methiocarb | 0.01 mg/kg |  | |  |  |
| Isopropalin | 0.005 mg/kg | Methiocarb-sulfon | 0.01 mg/kg |  | |  |  |
| Isoxadifen-ethyl | 0.01 mg/kg | Methiocarb-sulfoxid | 0.01 mg/kg |  | |  |  |
| Kresoxim-methyl | 0.01 mg/kg | Methomyl | 0.01 mg/kg |  | |  |  |
| Leptophos | 0.01 mg/kg | Methoprotryn | 0.01 mg/kg |  | |  |  |
| Lindan | 0.005 mg/kg | Methoxyfenozid | 0.01 mg/kg |  | |  |  |
| Lufenuron | 0.02 mg/kg | Metobromuron | 0.01 mg/kg |  | |  |  |
| Malaoxon | 0.02 mg/kg | Metolachlor | 0.01 mg/kg |  | |  |  |
| Malathion | 0.005 mg/kg | Metolcarb | 0.01 mg/kg |  | |  |  |
| Mecarbam | 0.01 mg/kg | Metoxuron | 0.01 mg/kg |  | |  |  |
| Mephosfolan | 0.01 mg/kg | Metrafenon | 0.01 mg/kg |  | |  |  |
| Merphos | 0.01 mg/kg | Metribuzin | 0.01 mg/kg |  | |  |  |
| Metazachlor | 0.1 mg/kg | Metsulfuron-methyl | 0.01 mg/kg |  | |  |  |
| Methacrifos | 0.025 mg/kg | Molinat | 0.01 mg/kg |  | |  |  |
| Methidathion | 0.02 mg/kg | Monocrotophos | 0.01 mg/kg |  | |  |  |
| Methoxychlor | 0.05 mg/kg | Monolinuron | 0.01 mg/kg |  | |  |  |
| Metolachlor | 0.1 mg/kg | Monuron | 0.01 mg/kg |  | |  |  |
| Metrafenon | 0.01 mg/kg | Napropamid | 0.01 mg/kg |  | |  |  |
| Metribuzin | 0.01 mg/kg | Neburon | 0.01 mg/kg |  | |  |  |
| Mirex | 0.01 mg/kg | Novaluron | 0.02 mg/kg |  | |  |  |
| Molinat | 0.1 mg/kg | Nuarimol | 0.01 mg/kg |  | |  |  |
| Myclobutanil | 0.01 mg/kg | Ofurace | 0.01 mg/kg |  | |  |  |
| Nitralin | 0.01 mg/kg | Omethoat | 0.01 mg/kg |  | |  |  |
| Nitrapyrin | 0.02 mg/kg | Orbencarb | 0.01 mg/kg |  | |  |  |
| Nitrofen | 0.01 mg/kg | Oxadixyl | 0.01 mg/kg |  | |  |  |
| Nitrothal-isopropyl | 0.02 mg/kg | Oxamyl | 0.01 mg/kg |  | |  |  |
| Norflurazon | 0.025 mg/kg | Oxamyl-oxim | 0.01 mg/kg |  | |  |  |
| Nuarimol | 0.01 mg/kg | Oxydemeton-methyl | 0.01 mg/kg |  | |  |  |
| Oxadiazon | 0.01 mg/kg | Paclobutrazole | 0.01 mg/kg |  | |  |  |
| Oxydemeton-methyl | 0.02 mg/kg | Paraoxon-ethyl | 0.01 mg/kg |  | |  |  |
| Oxyfluorfen | 0.005 mg/kg | Paraoxon-methyl | 0.01 mg/kg |  | |  |  |
| Paclobutrazole | 0.01 mg/kg | Penconazol | 0.01 mg/kg |  | |  |  |
| Paraoxon-ethyl | 0.02 mg/kg | Pencycuron | 0.01 mg/kg |  | |  |  |
| Paraoxon-methyl | 0.02 mg/kg | Pendimethalin | 0.01 mg/kg |  | |  |  |
| Parathion-ethyl | 0.01 mg/kg | Phenmedipham | 0.01 mg/kg |  | |  |  |
| Parathion-methyl | 0.01 mg/kg | Phorat | 0.05 mg/kg |  | |  |  |
| PCB IUPAC - Nr. 52 | 0.01 mg/kg | Phorate-sulfon | 0.01 mg/kg |  | |  |  |
| PCB IUPAC - Nr. 101 | 0.01 mg/kg | Phorate-sulfoxid | 0.01 mg/kg |  | |  |  |
| PCB IUPAC - Nr. 138 | 0.005 mg/kg | Phosmet | 0.05 mg/kg |  | |  |  |
| PCB IUPAC - Nr. 153 | 0.005 mg/kg | Phosphamidon | 0.01 mg/kg |  | |  |  |
| PCB IUPAC - Nr. 180 | 0.005 mg/kg | Phoxim | 0.01 mg/kg |  | |  |  |
| Penconazol | 0.01 mg/kg | Picoxystrobin | 0.01 mg/kg |  | |  |  |
| Pendimethalin | 0.01 mg/kg | Piperonylbutoxid | 0.01 mg/kg |  | |  |  |
| Pentachloranilin | 0.01 mg/kg | Pirimicarb | 0.01 mg/kg |  | |  |  |
| Pentachlorbenzol | 0.005 mg/kg | Pirimicarb, desmethyl- | 0.01 mg/kg |  | |  |  |
| Permethrin | 0.02 mg/kg | Pirimicarb, desmethyl-formamido- | 0.05 mg/kg |  | |  |  |
| Perthan | 0.1 mg/kg | Primisulfuron-methyl | 0.02 mg/kg |  | |  |  |
| Phenkapton | 0.01 mg/kg | Prochloraz | 0.01 mg/kg |  | |  |  |
| Phenothrin | 0.05 mg/kg | Promecarb | 0.01 mg/kg |  | |  |  |
| Phenthoat | 0.01 mg/kg | Prometon | 0.01 mg/kg |  | |  |  |
| Phosalon | 0.01 mg/kg | Prometryn | 0.01 mg/kg |  | |  |  |
| Phosfolan | 0.01 mg/kg | Propamocarb | 0.01 mg/kg |  | |  |  |
| Phosmet | 0.01 mg/kg | Propargit | 0.01 mg/kg |  | |  |  |
| Picolinafen | 0.01 mg/kg | Propazin | 0.01 mg/kg |  | |  |  |
| Picoxystrobin | 0.01 mg/kg | Propham | 0.05 mg/kg |  | |  |  |
| Piperophos | 0.05 mg/kg | Propiconazol | 0.01 mg/kg |  | |  |  |
| Pirimiphos-ethyl | 0.01 mg/kg | Propoxur | 0.01 mg/kg |  | |  |  |
| Pirimiphos-methyl | 0.01 mg/kg | Propoxycarbazon | 0.02 mg/kg |  | |  |  |
| Plifenat | 0.01 mg/kg | Proquinazid | 0.01 mg/kg |  | |  |  |
| Prallethrin | 0.02 mg/kg | Prosulfocarb | 0.01 mg/kg |  | |  |  |
| Procymidon | 0.04 mg/kg | Prosulfuron | 0.01 mg/kg |  | |  |  |
| Profenofos | 0.005 mg/kg | Pymetrozin | 0.01 mg/kg |  | |  |  |
| Profluralin | 0.005 mg/kg | ***Pyraclostrobin*** | 0.01 mg/kg |  | |  |  |
| Propachlor | 0.1 mg/kg | Pyraflufen-ethyl | 0.01 mg/kg |  | |  |  |
| Propanil | 0.01 mg/kg | Pyridat | 0.05 mg/kg |  | |  |  |
| Propazin | 0.05 mg/kg | ***Pyrimethanil*** | 0.01 mg/kg |  | |  |  |
| Propetamphos | 0.005 mg/kg | Pyrimidifen | 0.01 mg/kg |  | |  |  |
| Propiconazol | 0.01 mg/kg | Pyriproxyfen | 0.01 mg/kg |  | |  |  |
| Propyzamid | 0.02 mg/kg | Quizalofop-ethyl | 0.01 mg/kg |  | |  |  |
| Prothiofos | 0.005 mg/kg | Rabenzazol | 0.01 mg/kg |  | |  |  |
| Prothoat | 0.01 mg/kg | Rimsulfuron | 0.05 mg/kg |  | |  |  |
| Pyraflufen-ethyl | 0.02 mg/kg | Sebuthylazin | 0.01 mg/kg |  | |  |  |
| Pyrazophos | 0.01 mg/kg | Sethoxydim | 0.01 mg/kg |  | |  |  |
| Pyrethrine | 0.02 mg/kg | Siduron | 0.01 mg/kg |  | |  |  |
| Pyridaben | 0.01 mg/kg | Silafluofen | 0.02 mg/kg |  | |  |  |
| Pyridaphenthion | 0.01 mg/kg | Simazin | 0.01 mg/kg |  | |  |  |
| Pyrifenox | 0.02 mg/kg | Simazin, desethyl- | 0.01 mg/kg |  | |  |  |
| Pyrimitat | 0.05 mg/kg | Simeconazol | 0.01 mg/kg |  | |  |  |
| Quinalphos | 0.005 mg/kg | Spinosad | 0.02 mg/kg |  | |  |  |
| Quinoxyfen | 0.02 mg/kg | Spirodiclofen | 0.01 mg/kg |  | |  |  |
| Quintozen | 0.005 mg/kg | Spiromesifen | 0.01 mg/kg |  | |  |  |
| Quizalofop-ethyl | 0.02 mg/kg | Spiroxamin | 0.01 mg/kg |  | |  |  |
| Resmethrin | 0.1 mg/kg | Sulfotepp | 0.01 mg/kg |  | |  |  |
| S 421 | 0.01 mg/kg | ***Tebuconazol*** | 0.01 mg/kg |  | |  |  |
| Spiromesifen | 0.02 mg/kg | Tebufenozid | 0.01 mg/kg |  | |  |  |
| Sulfotepp | 0.025 mg/kg | Tebufenpyrad | 0.01 mg/kg |  | |  |  |
| Sulprofos | 0.01 mg/kg | Teflubenzuron | 0.05 mg/kg |  | |  |  |
| Swep | 0.1 mg/kg | TEPP | 0.01 mg/kg |  | |  |  |
| tau-Fluvalinat | 0.005 mg/kg | Terbacil | 0.01 mg/kg |  | |  |  |
| Tebupirimfos | 0.01 mg/kg | Terbufos | 0.01 mg/kg |  | |  |  |
| Tecnazen | 0.005 mg/kg | Terbufos-sulfon | 0.01 mg/kg |  | |  |  |
| Tefluthrin | 0.005 mg/kg | Terbufos-sulfoxid | 0.01 mg/kg |  | |  |  |
| Temephos | 0.01 mg/kg | Terbuthylazin | 0.01 mg/kg |  | |  |  |
| Terbufos | 0.005 mg/kg | Terbuthylazin, desethyl- | 0.01 mg/kg |  | |  |  |
| Tetrachlorvinphos | 0.005 mg/kg | Terbutryn | 0.01 mg/kg |  | |  |  |
| Tetraconazol | 0.01 mg/kg | Tetraconazol | 0.01 mg/kg |  | |  |  |
| Tetradifon | 0.005 mg/kg | Thiabendazol | 0.01 mg/kg |  | |  |  |
| Tetramethrin | 0.01 mg/kg | Thiabendazol, 5-hydroxy- | 0.01 mg/kg |  | |  |  |
| Tetrasul | 0.01 mg/kg | Thiacloprid | 0.01 mg/kg |  | |  |  |
| Tolclofos-methyl | 0.005 mg/kg | Thiametoxam | 0.01 mg/kg |  | |  |  |
| Tolylfluanid | 0.01 mg/kg | Thiametoxam/Clothianidin (Summe) | 0.01 mg/kg |  | |  |  |
| ToxaphenParlar-Nr. 26 (Indikatorverbindung) | 0.01 mg/kg | Thiazafluron | 0.01 mg/kg |  | |  |  |
| ToxaphenParlar-Nr. 50 (Indikatorverbindung) | 0.01 mg/kg | Thifensulfuron-methyl | 0.01 mg/kg |  | |  |  |
| ToxaphenParlar-Nr. 62 (Indikatorverbindung) | 0.01 mg/kg | Thiocarbazil | 0.01 mg/kg |  | |  |  |
| Transfluthrin | 0.01 mg/kg | Thiodicarb | 0.01 mg/kg |  | |  |  |
| Triadimefon | 0.01 mg/kg | Thiofanox | 0.05 mg/kg |  | |  |  |
| Triadimenol | 0.1 mg/kg | Thiofanox-sufon | 0.01 mg/kg |  | |  |  |
| Triallat | 0.01 mg/kg | Thiometon | 0.05 mg/kg |  | |  |  |
| Triamiphos | 0.025 mg/kg | Thionazin | 0.01 mg/kg |  | |  |  |
| Triazophos | 0.02 mg/kg | Thiophanat (-ethyl) | 0.01 mg/kg |  | |  |  |
| Tribufos | 0.005 mg/kg | Thiophanat-methyl | 0.01 mg/kg |  | |  |  |
| Trichloronat | 0.005 mg/kg | Triadimefon | 0.01 mg/kg |  | |  |  |
| Tridiphan | 0.05 mg/kg | Triadimenol | 0.01 mg/kg |  | |  |  |
| Trifloxystrobin | 0.01 mg/kg | Triamiphos | 0.01 mg/kg |  | |  |  |
| Trifluralin | 0.005 mg/kg | Triasulfuron | 0.01 mg/kg |  | |  |  |
| Vamidothion | 0.025 mg/kg | Triazamat | 0.01 mg/kg |  | |  |  |
| Vinclozolin | 0.005 mg/kg | Triazophos | 0.01 mg/kg |  | |  |  |
|  |  | Tribenuron-methyl | 0.01 mg/kg |  | |  |  |
|  |  | Trichlorfon | 0.02 mg/kg |  | |  |  |
|  |  | Tricyclazol | 0.01 mg/kg |  | |  |  |
|  |  | Tridemorph | 0.1 mg/kg |  | |  |  |
|  |  | Trietazin | 0.01 mg/kg |  | |  |  |
|  |  | Trifloxystrobin | 0.01 mg/kg |  | |  |  |
|  |  | Trifloxysulfuron | 0.01 mg/kg |  | |  |  |
|  |  | Triflumizol | 0.01 mg/kg |  | |  |  |
|  |  | Triflumuron | 0.01 mg/kg |  | |  |  |
|  |  | Triflusulfuron-methyl | 0.05 mg/kg |  | |  |  |
|  |  | Triforin | 0.01 mg/kg |  | |  |  |
|  |  | Trimethacarb, 3,4,5- | 0.01 mg/kg |  | |  |  |
|  |  | Triticonazol | 0.01 mg/kg |  | |  |  |
|  |  | Uniconazol | 0.01 mg/kg |  | |  |  |
|  |  | Vamidothion | 0.01 mg/kg |  | |  |  |
|  |  | Vamidothion-sulfon | 0.01 mg/kg |  | |  |  |
|  |  | Vamidothion-sulfoxid | 0.01 mg/kg |  | |  |  |
|  |  | Zoxamid | 0.01 mg/kg |  | |  |  |
|  |  |  |  |  | |  |  |
