# Supplementary material for: Health status of free-ranging ring-necked pheasant chicks (Phasianus colchicus) in North-Western Germany
Source: PLoS One. 2020 Jun 16;15(6):e0234044. doi: 10.1371/journal.pone.0234044 (PMC7297342; doi:10.1371/journal.pone.0234044)
Supplement: S1 Data — (PDF) [file pone.0234044.s002.pdf]

ID	Altersklasse	Bundesland	Landkreis	Gemeinde
1	2	NI	Osnabrück	Neuenkirchen
2	2	NI	Osnabrück	Neuenkirchen
3	2	NI	Osnabrück	Neuenkirchen
4	1	NI	Emsland	Meppen
5	1	NI	Emsland	Meppen
6	2	NI	Grafschaft Bentheim	Wilsum
7	2	NI	Cloppenburg	Strücklingen
8	2	NI	Emsland	Meppen
9	2	NI	Emsland	Meppen

10	2	NI	Emsland	Meppen
11	2	NI	Emsland	Meppen
12	3	NI	Grafschaft Bentheim	Wilsum
13	3	NI	Grafschaft Bentheim	Wilsum
14	3	NI	Cuxhaven	Osten
15	3	NI	Cuxhaven	Osten
16	3	NRW		Ahlen

17	2	NRW		Ahlen
18	3	NRW		Ahlen
19	3	NRW		Ahlen
20	3	NI	Cuxhaven	Hemmoor
21	3	NI	Cuxhaven	Hemmoor
22	3	NI	Cuxhaven	Hemmoor
23	2	NI	Cuxhaven	Hemmoor
24	2	NI	Emsland	Meppen
25	3	NI	Emsland	Meppen
26	3	NI	Emsland	Meppen
27	3	NI	Osnabrück	Neuenkirchen
28	3	NRW	Coesfeld	Dülmen

29	3	NRW	Coesfeld	Dülmen
30	3	NRW	Coesfeld	Dülmen
31	3	NRW	Coesfeld	Dülmen
32	2	NRW		Lippstadt
33	3	SH		
34	3	SH		
35	2	NRW	Coesfeld	Dülmen
36	2	NRW	Coesfeld	Dülmen
37	2	NRW	Coesfeld	Dülmen
38	3	NRW	Coesfeld	Dülmen
39	1	NRW	Warendorf	Ahlen
40	1	NRW	Warendorf	Ahlen
41	1	NRW	Warendorf	Ahlen
42	1	NRW	Warendorf	Ahlen
43	1	NRW	Warendorf	Ahlen
44	1	NRW	Warendorf	Ahlen

45	1	NRW	Warendorf	Ahlen
46	1	NRW	Warendorf	Ahlen
47	1	NRW	Warendorf	Ahlen
48	1	NRW	Warendorf	Ahlen
49	1	NRW	Warendorf	Ahlen
50	1	NRW	Warendorf	Ahlen
51	3	NI	Vechta	Vechta
52	3	NI	Emsland	Meppen
53	3	NI	Emsland	Meppen
54	3	NI	Vechta	Vechta
55	3	NRW	Coesfeld	Welte
56	3	NRW	Coesfeld	Welte
57	3	SH	Dithmarschen	Warwerort
58	3	SH	Dithmarschen	Warwerort
59	3	SH	Dithmarschen	Warwerort

60	3	SH	Dithmarschen	Warwerort
61	3	NI	Emsland	Meppen
62	3	NI	Grafschaft Bentheim	Wilsum

geschätztes Alter	Gewicht in kg	Geschlecht	Ernährungszustand	Syngamus	Endoparasiten laut Patho
4 Wochen	0,11	w	mäßig	neg	neg
4 Wochen	0,12	w	mäßig	neg	hgr Kokzidien
4 Wochen	0,11	w	mäßig	neg	mgr Kokzidien
3 Wochen	0,08	w	mäßig	neg	neg
2-3 Wochen	0,08	w	mäßig	neg	Kokzidien
(4-)5 Wochen	0,15	w	gut	neg	neg
	0,19	w	gut	neg	neg
5 Wochen	0,18	w	gut	neg	neg
knapp 5 Wochen	0,11	w	mäßig	neg	hgr Kokzidien

5 Wochen	0,15	w	gut	neg	neg
5 Wochen	0,15	w	mäßig	neg	neg
7 Wochen	0,34	m	gut	neg	neg
7 Wochen	0,36	m	gut	neg	neg
7-8 Wochen	0,25	w	schlecht	neg	neg
7-8 Wochen	0,27	w	mäßig	neg	neg
6-7 Wochen	0,27	m	mäßig	mgr	neg

(3-) 4 Wochen (Wechselt gerade H1 -> mit 27 Tagen)	0,1	m	gut	neg	neg
(6-) 7 Wochen	0,28	w	gut	neg	neg
7 Wochen	0,37	m	gut	hgr	neg
7 Wochen	0,35	w	gut	neg	Syngamus trachea; Kokzidien
7 - 8 Wochen	0,4	m	gut	neg	Syngamus
7 - 8 Wochen	0,42	m	gut	neg	ggr Kokzidien; Nematoden; Protozoen, Syngamus
4-5 Wochen	0,18	m	gut	neg	ggr Kokzidien, Syngamus
(5-) 6 Wochen	0,31	w	mäßig	neg	neg
8 bis 9 Wochen	0,48	w	mäßig	neg	hgr Protozoäre Erreger; hgr Nematodeneier
8 bis 9 Wochen	0,5	m	mäßig	neg	hgr Protozoäre Erreger
(9 bis) 11 Wochen	0,5	w	gut bis mäßig	neg	neg
knapp 7 Wochen	0,416	m	gut	ggr	neg

knapp 7 Wochen	0,455	m	gut	neg	neg
(7 bis) 8 Wochen	0,396	w	gut	ggr	neg
8 bis 9 Wochen	0,435	w	gut	neg	ggr Kokzidien, Syngamus
5 bis 6 Wochen	0,18	w	mäßig	neg	hgr Kokzidien, Nematodeneier
Messung nicht eindeutig…, waren fast erwachsen	0,75	w	gut	neg	neg
Messung nicht eindeutig…	0,94	m	mäßig	neg	solitärer Nematode
(5 bis) 6 Wochen	0,24	m	gut	mgr	neg
etwa 6 Wochen	0,26	w	gut	neg	hgr Syngamus
etwa 6 Wochen	0,27	w	gut	mgr	neg
8 bis 10 Wochen	0,43	w	gut	neg	hgr Syngamus trachea
1-2 Tage	0,06	w	mäßig bis schlecht	neg	ggr Kokzidien
1-2 Tage	0,05	m	mäßig bis schlecht	neg	hgr Kokzidien
1-2 Tage	0,02	?	mäßig	neg	neg
1-2 Tage	0,02	?	mäßig	neg	neg
1-2 Tage	0,02	?	mäßig	neg	neg
1-2 Tage	0,02	?	mäßig	neg	neg

1-2 Tage	0,02	?	mäßig	neg	neg
1-2 Tage	0,02	?	mäßig	neg	neg
1-2 Tage	0,02	?	mäßig	neg	neg
1-2 Tage	0,02	?	mäßig	neg	neg
1-2 Tage	0,02	?	mäßig	neg	neg
1-2 Tage	0,02	?	mäßig	neg	neg
		w	gut	ggr	hgr Schizonten von Protozoen
	0,297	m	gut	neg	neg
	0,28	w	gut	neg	neg
	0,78	w	gut	neg	neg
	0,87	m	gut	neg	neg
	0,78	m	gut	neg	neg
	0,62	m	gut	neg	neg
	0,47	w	gut	ggr	neg
	0,56	w	gut	neg	neg

0,61	m	gut	ggr	neg
0,29	w	gut	neg	neg
0,87	w	gut	neg	ggr Kokzidien

Ektoparasiten	Herpes-PCR	aMPV-PCR	IBDV-PCR	Corona PCR	Corona Anzucht
neg	n.u.	neg	neg	Zt/Tr neg	n.u.
neg	n.u.	neg	neg	Zt/Tr neg	n.u.
neg	n.u.	neg	neg	Zt/Tr neg	n.u.
neg	ILT neg	neg	neg	Zt/Tr neg	n.u.
neg	n.u.	neg	neg	Zt/Tr neg	n.u.
neg	n.u.	neg	neg	fehlt	n.u.
neg	n.u.	neg	neg	fehlt	n.u.
neg	n.u.	neg	neg	Tr neg	n.u.
neg	n.u.	neg	neg	Tr neg	n.u.

neg	n.u.	neg	neg	fehlt	n.u.
neg	ILT neg	neg	neg	fehlt	n.u.
neg	n.u.	neg	neg	Tr neg	n.u.
neg	n.u.	neg	neg	Tr neg	n.u.
neg	ILT neg	neg	neg	Tr neg	n.u.
neg	n.u.	neg	neg	Tr neg	n.u.
neg	ILT neg	neg	neg	fehlt	n.u.

neg	ILT neg	neg	neg	Tr neg	n.u.
neg	n.u.	neg	neg	Tr neg	n.u.
neg	n.u.	neg	neg	Tr neg	n.u.
neg	n.u.	neg	neg	Tr neg	n.u.
neg	n.u.	neg	neg	Tr neg	n.u.
neg	ILT neg	neg	neg	Tr neg	n.u.
neg	n.u.	neg	neg	Tr neg	n.u.
neg	n.u.	neg	neg	Tr neg	n.u.
neg	ILT neg	neg	neg	Tr neg	n.u.
Ixodes sp	ILT neg	neg	neg	Tr neg	n.u.
neg	ILT neg	neg	neg	Tr neg	n.u.
Federlinge	n.u.	neg	neg	Zt/Tr neg	n.u.

Federlinge	ILT neg	neg	neg	Tr neg	n.u.
Federlinge	n.u.	neg	neg	Tr neg	n.u.
Federlinge	n.u.	neg	neg	Zt/Tr neg	n.u.
Federlinge	n.u.	neg	neg	Zt/Tr neg	n.u.
neg	n.u.	neg	neg	Zt/Tr neg	n.u.
neg	n.u.	neg	neg	Zt/Tr neg	n.u.
neg	neg	neg	neg	Tr neg	n.u.
Federlinge	n.u.	neg	neg	Tr neg	n.u.
Federlinge	n.u.	neg	neg	Tr neg	n.u.
neg	n.u.	neg	neg	Tr neg	n.u.
neg	n.u.	n.u.	n.u.	n.u.	n.u.
neg	n.u.	n.u.	n.u.	n.u.	n.u.
neg	n.u.	n.u.	n.u.	n.u.	n.u.
neg	n.u.	n.u.	n.u.	n.u.	n.u.
Ixodes sp	n.u.	n.u.	n.u.	n.u.	n.u.
neg	n.u.	n.u.	n.u.	n.u.	n.u.

neg	n.u.	n.u.	n.u.	n.u.	n.u.
neg	n.u.	n.u.	n.u.	n.u.	n.u.
neg	n.u.	n.u.	n.u.	n.u.	n.u.
neg	n.u.	n.u.	n.u.	n.u.	n.u.
neg	n.u.	n.u.	n.u.	n.u.	n.u.
neg	n.u.	n.u.	n.u.	n.u.	n.u.
neg	n.u.	n.u.	n.u.	n.u.	n.u.
hgr Federlinge	n.u.	n.u.	n.u.	n.u.	n.u.
neg					
neg	n.u.	n.u.	n.u.	n.u.	n.u.
neg					
neg					
neg					
neg					
neg					

neg

neg

neg

Siadeno PCR
neg
neg
neg
neg
neg
neg
neg
neg
neg

neg

neg

neg

neg

neg

neg

neg

neg

neg

neg

neg

neg

neg

neg

neg

neg

neg

neg

neg

neg

neg

neg

k.P.

neg

neg

neg

neg

neg

neg

n.u.

n.u.

n.u.
n.u.

n.u.
n.u.

n.u.

n.u.

n.u.

n.u.

n.u.

n.u.

n.u.

n.u.

n.u.
