# Supplementary material for: Health status of free-ranging ring-necked pheasant chicks (Phasianus colchicus) in North-Western Germany
Source: PLoS One. 2020 Jun 16;15(6):e0234044. doi: 10.1371/journal.pone.0234044 (PMC7297342; doi:10.1371/journal.pone.0234044)
Supplement: Attachment — Submitted filename: Review journal notes.docx [file pone.0234044.s003.docx]

**Writing style:**

- Inconsistent writing
- Grammatical issues (sentences not necessarily making sense or flowing nicely, not very easy to read in some cases)
- Flow from one point to another is lacking (jumps from one point to next)
- Few writing errors (no gaps between full stops, random letters throughout paragraphs)
- Sometimes no gaps between paragraphs (doesn’t look professional) and isn’t consistent with the rest of the document
- Inconsistent with giving data and results (sixteen in 54, 12 in 67 – should stick to one format etc.)
- Inconsistent explanations for doing something or none at all

**Questions:**

1. What acid and alkaline-free derivative substances?
2. What other factors may weaken the population?
3. Why focus on birds up to 11 weeks of age?
4. Exactly how many chicks were taken? (at maximum half isn’t an amount unless you first specify the amount caught)
5. Why did the animals have to die for you to test this?
6. Nutritional condition score previously described where?

**Comments:**

Don’t agree with methodology – seems counterintuitive and backwards to what they opened the paper with, and the decline was the entire reason behind the study.
